# Structure-Dependent Innate Immune Compatibility of Sup35 G7-Derived Self-Assembling Peptide Nanomaterials in an Immune-Compatibility Assessment

**DOI:** 10.3390/vaccines14090734

**Published:** 2026-08-25

**Authors:** Jinglin Song, Shixiong Shen, Langzhi He, Hasen Bilige, Chen Li, Yuedan Wang, Wenjun Ma, Yun Wang

**Affiliations:** 1 School of Public Health, Peking University Health Science Center, Beijing 100091, China; songjinglin@stu.pku.edu.cn (J.S.); helangzhi@bjmu.edu.cn (L.H.); 2311110199@bjmu.edu.cn (H.B.); 2311210084@stu.pku.edu.cn (C.L.); 2 Department of Anesthesiology, Peking University Third Hospital, Beijing 100191, China; shixiongshen@stu.pku.edu.cn; 3 NHC Key Laboratory of Medical Immunology (Peking University), Department of Immunology, School of Basic Medical Sciences, Peking University, Beijing 100191, China; wangyuedan@bjmu.edu.cn; 4 Beijing Key Laboratory of Toxicological Research and Risk Assessment for Food Safety, Peking Univesity, Beijing 100191, China

**Keywords:** self-assembling peptide, nanovaccine, nanomaterials, toxicity, immune cells

## Abstract

Objective: Self-assembling peptide nanomaterials are increasingly investigated as modular platforms for vaccine delivery and immune modulation. However, their structure-dependent interactions with immune cells remain insufficiently defined, limiting their early evaluation as peptide nanomaterial. This study evaluated the physicochemical properties and immunological effects of three Sup35 G7-derived self-assembling peptide nanomaterials, G7, G7d, and G7d(PEG)HER2, with particular attention to innate immune compatibility and macrophage responses. Methods: The morphology and surface charge of the peptides were characterized by transmission electron microscopy and zeta-potential analysis. Their cytocompatibility was assessed in THP-1 macrophages, Jurkat T cells, and IM-9 B cells. Transcriptomic and proteomic analyses were integrated to identify immune-cell-specific molecular responses. Cellular uptake, apoptosis, cytokine secretion, and intracellular reactive oxygen species production were further examined in macrophages. In vivo immunization in BALB/c mice was conducted to assess systemic immunoglobulin responses. Results: G7 and G7d assembled into large crystalline aggregates with limited dispersibility, whereas PEGylated G7d(PEG)HER2 formed more uniform nanofibers. All three materials showed positive zeta potentials and relatively low cytotoxicity across the tested immune cell models. Among these cells, macrophages displayed the strongest molecular responses. Integrated transcriptomic and proteomic analyses revealed coordinated alterations mainly involving metabolic remodeling, redox homeostasis, proteasome activity, and mitochondrial-associated pathways, rather than predominant activation of classical pro-inflammatory signaling. Ultrastructural analysis confirmed intracellular uptake of assembled peptides and mitochondrial morphological changes without detectable apoptosis. Cytokine profiling showed reduced secretion of multiple cytokines and chemokines, while G7 induced higher intracellular reactive oxygen species than G7d and G7d(PEG)HER2. In vivo, G7d administration was associated with increased serum IgG levels, whereas G7 showed a moderate effect and G7d(PEG)HER2 exhibited minimal changes; IgM levels remained unchanged. Conclusions: Sup35 G7 based self-assembling peptides exhibit low acute toxicity but induce structure-dependent modulation of macrophage function. Differences in assembly morphology and surface modification may influence systemic humoral responses. These findings support the importance of physicochemical design in evaluating immune compatibility of peptide nanomaterial scaffolds.

## 1. Introduction

Self-assembling peptide nanomaterials (SAPs) are ordered supramolecular nanostructures formed through the spontaneous organization of short peptides or polypeptides driven by non-covalent interactions, including hydrogen bonding, hydrophobic interactions, π–π stacking, and electrostatic forces [1]. Under defined environmental conditions, such as pH, temperature, ionic strength, and peptide concentration, peptide building blocks can autonomously assemble into nanostructures with distinct morphologies and biological functions [2]. Peptide sequence, chirality, secondary structure, and chemical modification are key determinants of self-assembly pathways and resulting nanoscale architectures, enabling the rational design of functional materials for drug delivery, tissue engineering, biosensing, immunotherapy, and vaccine-related applications [2]. In the context of peptide-based vaccine design, self-assembled peptide structures are of particular interest because they may serve as antigen carriers, depot-forming materials, multivalent display platforms, or immunomodulatory scaffolds [3]. However, the same physicochemical features that support their biomedical utility, including size, morphology, aggregation behavior, and surface charge, may also determine their interaction with immune cells and thereby influence both immunogenicity and safety.

Effective vaccination depends on coordinated communication between innate and adaptive immunity. Innate immune cells, including macrophages, dendritic cells, and natural killer cells, rapidly detect exogenous or danger-associated signals through pattern recognition receptors (PRRs), thereby mediating phagocytosis, cytotoxic responses, cytokine release, and inflammatory regulation during early immune activation [4]. These innate immune processes provide essential signals for the initiation and shaping of adaptive immune responses [5]. Dendritic cells, as professional antigen-presenting cells (APCs), bridge innate and adaptive immunity by processing antigens, presenting peptide fragments via major histocompatibility complex (MHC) molecules, and delivering co-stimulatory signals required for T-cell priming [6]. Macrophages also play important roles in particle uptake, inflammatory regulation, tissue surveillance, and antigen handling. Adaptive immune cells, primarily T and B lymphocytes, subsequently execute antigen-specific cellular and humoral immune responses [7]. Therefore, understanding how candidate vaccine-related nanomaterials interact with macrophages, T cells, and B cells is essential for evaluating their immune compatibility before further translational development.

Peptide-based nanotherapeutic systems have been actively investigated because peptide sequences can be rationally designed to confer targeting, membrane interaction, organelle localization, or stimulus-responsive functions. For example, ALM201, with the peptide sequence IRQQPRDPPTETLELEVSPDPAS, selectively binds to the tumor vascular endothelial protein FKBPL, thereby inhibiting angiogenesis and reducing cancer stem cell populations [8]. A CD44-targeted polymer–peptide–doxorubicin conjugate, A6–PS–EPI, uses the UP based A6 peptide (KPSSPPEE) to recognize CD44 and anchor cytotoxic agents to tumor cells, followed by pH-responsive intracellular drug release [9,10]. The amphipathic cationic peptide KLAK(KLAKLAK)_2_ preferentially associates with negatively charged mitochondrial membranes, disrupts membrane integrity, and induces apoptosis [11,12]. Melittin, a bee venom based amphipathic α-helical peptide, interacts with phospholipid bilayers and permeabilizes membranes through pore formation [13]. Other engineered peptide constructs, such as CNGRC-GG-D(KLAKLAK)_2_, PpIX–PEG–(KLAKLAK)_2_, and reactive oxygen species-responsive thioketal–PEG–KLVFF–KLAK nanoparticles, further demonstrate that peptide sequence and supramolecular morphology can determine cellular targeting, intracellular delivery, mitochondrial perturbation, and oxidative stress responses [11,14,15]. These studies collectively highlight the importance of defining structure–function relationships when peptide nanomaterials are considered for immune-related biomedical applications.

For vaccine delivery applications, peptide nanomaterials should ideally combine favorable structural stability with predictable immune-cell interactions [16]. Excessive cytotoxicity or uncontrolled inflammatory activation may compromise safety, whereas insufficient interaction with innate immune cells may limit antigen delivery or immune modulation [17]. In particular, macrophage responses are highly relevant because macrophages are efficient in particle recognition and internalization and can shape the cytokine milieu that influences downstream adaptive immunity [18]. Surface charge, morphology, aggregation state, and chemical modification, including PEGylation, may alter cellular uptake, intracellular trafficking, redox homeostasis, mitochondrial function, and cytokine secretion [19]. Therefore, systematic evaluation of these parameters is required to support the safety-oriented design of self-assembling peptide nanomaterials as candidate peptide nanomaterials.

Sup35 G7 based peptides represent a useful model for studying how peptide assembly structure and surface modification influence immune-cell responses. In this study, we selected three SUP35 G7 based self-assembling peptide nanomaterials with distinct physicochemical properties, including G7, G7d, and G7d(PEG)HER2. We systematically characterized their assembly morphology and surface charge and evaluated their effects on representative human immune cell models, including THP-1 based macrophages, Jurkat T cells, and IM-9 B cells. Integrated transcriptomic and proteomic analyses were further performed to identify cell-type-specific molecular responses, with functional validation focusing on macrophage uptake, apoptosis, cytokine secretion, and reactive oxygen species production. By linking physicochemical properties with immune-cell responses, this study aims to define the innate immune compatibility and structure-dependent immunomodulatory features of SUP35 G7-derived SAPs, thereby providing a scientific basis for the safety evaluation of peptide nanomaterial.

## 2. Materials and Methods

### 2.1. Materials

All the peptides including G7 (Gly-Asn-Asn-Gln-Gln-Asn-Tyr), G7^d^ (DGly-DAsn-DAsn-DGln-DGln-DAsn-DTyr), G7^d^(PEG)HER2(Ac-DLys-His-His-His-Gly-Asn-Asn-Gln-DGln-Asn-Tyr-PEG4-DTyr-Cys-Asp-Gly-Phe-DTyr-Ala-Cys-DTyr-Met-Asp-Val-NH2) were designed by National Center for Nanoscience and Technology, and were synthesis by Chinese Peptide Company. All three peptides’ purity exceeds 95%, with an endotoxin level below 5 EU/mg.

### 2.2. Nanomaterial Characterization

The size and morphology of the peptides before and after self-assemble at different concentration is characterized by transmission electron microscopy (TEM) (JEOL, JEM-1400PLUS, Kyoto, Japan) at 100 kV. Peptides dissolved in PBS (100 μM, 500 μM, 1000 μM at 0 h, 0.5 h and 24 h) were applied to freshly glow-discharged carbon 200 mesh copper grids for 2 min and blotted with filter paper. Samples were stained with 2% uranyl acetate for 10 s and the blotted and air-dried. Grids were imaged in TEM and particle was analyzed and measured by EMSIS RADIUS Imaging Software (RADIUS 2.3).

The zeta potential of the peptides were measured using a Malvern Nano ZS Zetasizer (NanoSeries Zen 4003) (Malvern Instruments, Malvern, UK). The laser wavelength was set at 633 nm, and the angle between the incident and scattered light was 90°. All measurements were conducted at 25 °C. Each sample was analyzed for 20 cycles per run, and measurements were performed in triplicate.

### 2.3. Cell Culture

We chose 3 cell lines including: human monocytic leukmia cell line, THP-1 (SCSP-567, Procell, Wuhan, China), which is essential for differentiation into macrophages; human T lymphocyte cell line, Jurkat Clone E6-1 (SCSP-513, Procell); and human B lymphocyte cell line, IM-9 (CL-0367, Procell). The cell culture medium contained Rosewell Park Memorial Institute (RPMI) 1640 medium and supplemented with 10% (*v*/*v*) fetal bovine serum (FBS), 100 U mL^−1^ penicillin/streptomycin (P/S), and 10 mM N-(2-hydroxyethyl) piperazine-N′-ethanesulfonic acid (HEPES). At the same time, 37 °C and 5% CO_2_ maintained the cell growth. Phorbol 12-myristate 13 acetate (PMA, 10 ng mL^−1^) was added to the complete growth medium to promote the differentiated macrophages. All the medium were purchased from Procell.

### 2.4. Cell Viability Assay

THP-1 cells and Jurkat cells and IM-9 cells were cultured at the density of 4.0 × 10^5^ cells mL^−1^ (100 μL) in 96-well cell culture plates and THP-1 were differentiated with 10 ng mL^−1^ PMA for 48 h in a 5% CO_2_ humidified incubator at 37 °C. The differentiated macrophages, Jurkat and IM-9 were treated with G7, G7d and G7d(PEG)HER2 at 50 μM, 100 μM, 200 μM, 400 μM and 800 μM for 24 h. After washing twice (5 min/wash), 100 μL of fresh RPMI 1640 medium containing 10 μL of cell counting kit-8 (CCK-8) solution was added to each well of the plate and the absorbance was measured at 450 nm after 1 h incubation in a tissue culture incubator. The cell viability was calculated by cell viability (%) = [A (treatment) − A (blank)]/[A (control) − A (blank)] × 100%.

### 2.5. Omics Analysis

#### 2.5.1. Sample Collection and RNA Extraction

On the basis of the results of the viability test, we chose 500 μM G7 and G7d(PEG)HER2 as the research object. Each sample had three biological replicates. The samples were placed in an incubator filled with ice packs for temporary storage and then transported to the laboratory for RNA extraction and sequencing. Total RNA was extracted from cells using TRIzol^®^ reagent (Invitrogen, Carlsbad, CA, USA). The RNA concentration was subsequently determined via a spectrophotometer. The integrity of the RNA was detected using an Agilent 2100 Bioanalyzer (Agilent Technologies, Santa Clara, CA, USA).

#### 2.5.2. Transcriptome

Library preparation was performed first, followed by preliminary quantification using a Qubit 2.0 fluorometer. The samples were then detected using an Agilent 2100 bioanalyzer. If expected, the effective concentration was quantitatively analyzed via qRT-PCR followed by Illumina sequencing. Clean reads are remapped and the transcriptome is assembled using the RSEM method. The expression level of each gene was calculated using the FPKM method. Differential expression analysis was performed using the DESeq2 R package. When |log2(FoldChange)| ≥ 1 and *p*adj ≤ 0.05, it was assigned as the differential expression between two different conditions. All genes were annotated against the Eukaryotic Gene Ontology (GO) and the Kyoto Encyclopedia of Genes and Genomes (KEGG). For the enrichment analysis of DEGs, the GOseq R package and the KOBAS Web server were used to perform GO enrichment analysis and KEGG enrichment analysis of DEGs, respectively.

#### 2.5.3. Proteome

Proteomic analysis of the same samples used for transcriptome sequencing was performed by UHPLC. Differentially expressed proteins (DEPs) were defined as proteins with |log2(fold change)| > 2.0 and *p* < 0.05. Upregulated proteins were those with log2(fold change) > 2.0, whereas downregulated proteins were those with log2(fold change) < −2.0. Interproscan software 5.78-109.0 was used for GO functional annotation and KEGG functional protein family and pathway analyses of the DEPs.

### 2.6. Cellular Uptake of Self-Assembly Peptides

THP-1 cells treated by G7, G7d and G7d(PEG)HER2 at 500 μM concentration for 24 h, after fixation, rinsing, acetone graded dehydration, infiltration, embedding, polymerization, trimming, sectioning and grid staining, cell sections were imaged by TEM (JEOL, JEM-1400PLUS, Tokyo, Japan).

### 2.7. Cell Apoptosis Assay

THP-1 cells of 5 × 10^5^/mL were seeded onto six-well plates and incubated with G7, G7d, G7d(PEG)HER2 at 500 μM for 24 h. Cells were collected and washed with PBS. Then, resuspended cells in the binding buffer were stained with fluorescein isothiocyanate (FITC)–annexin V and Propidium Iodide Solution (PI) (Biolegend, San Diego, CA, USA) at room temperature for 15 min. A total of 1 × 10^4^ cells were collected and subjected to flow cytometry (BD Fortessa, San Jose, CA, USA), and acquired data were analyzed by FlowJo 10 software.

### 2.8. Change in Cytokines Level

The harvested cell culture media were centrifuged at 12,000 rpm at 10 min to remove any remaining particles. Macrophage cytokine release profiles including IL-1α, IL-1β, IL-6, IL-10, IL-12p70 were assessed by ProcartaPlex multiplex immunoassay (Thermo fisher, Carlsbad, CA, USA) according to the manufacturer’s instructions.

### 2.9. ROS Level Detection

THP-1 cells were incubated with G7, G7d and G7d(PEG)HER2 at 500 μM for 24 h. Then, cells were washed with PBS and incubated in 10 μM DCFH-DA (Beyotime, Shanghai, China) for 0.5 h at 37 °C. After incubation, cells were washed with PBS and analyzed using a ThermoFisher Varioskan Flash (Thermo fisher, Carlsbad, CA, USA).

### 2.10. In Vivo Immunization

Specific pathogen-free (SPF) female BALB/c mice (6–8 weeks old, 17–20 g) were obtained from the Department of Laboratory Animal Science, Peking University Health Science Center. During the experiment, mice were housed in an SPF barrier facility under controlled conditions, with a temperature of 20–25 °C, relative humidity of 40–70%, and a 12 h light/12 h dark cycle, with free access to food and water. All animal procedures complied with ethical standards for animal welfare and were approved by the Institutional Animal Care and Use Committee (Approval No. PUIRB-LA2022625).

After one week of acclimatization, mice were randomly divided into the following groups (n = 5 per group): control group, 20 mg/kg G7 group, G7d group, and G7d(PEG)HER2 group. Mice received subcutaneous injections in the dorsal neck region on day 0 (primary immunization), day 14 (booster immunization), and day 21 (enhanced immunization) with G7, G7d, or G7d(PEG)HER2 at doses of 20 mg/kg, respectively, while the control group received PBS. Three days after the final immunization, mice were sacrificed, and blood samples were collected.

### 2.11. Detection of Mice Serum Immunoglobulin Levels by Luminex

Blood samples were collected from mice and allowed to stand at room temperature for 2 h, followed by centrifugation at 1500× *g* for 20 min. Serum was then separated and aliquoted. The concentrations of IgG and IgM in mouse serum were determined using the ProcartaPlex™ Mouse Antibody Isotyping Panel (Thermo fisher, Carlsbad, CA, USA).

### 2.12. Data Analysis

All samples included three replicates. Analysis of variance (ANOVA) was performed on the data using SPSS 21.0. The data are expressed as the means ± standard errors. For multiple-group comparisons, one-way ANOVA followed by an appropriate post-hoc test should be applied to the replicate-level data before final statistical annotations are added to the figures.

## 3. Results

### 3.1. Material Characterization

**Figure 1:**
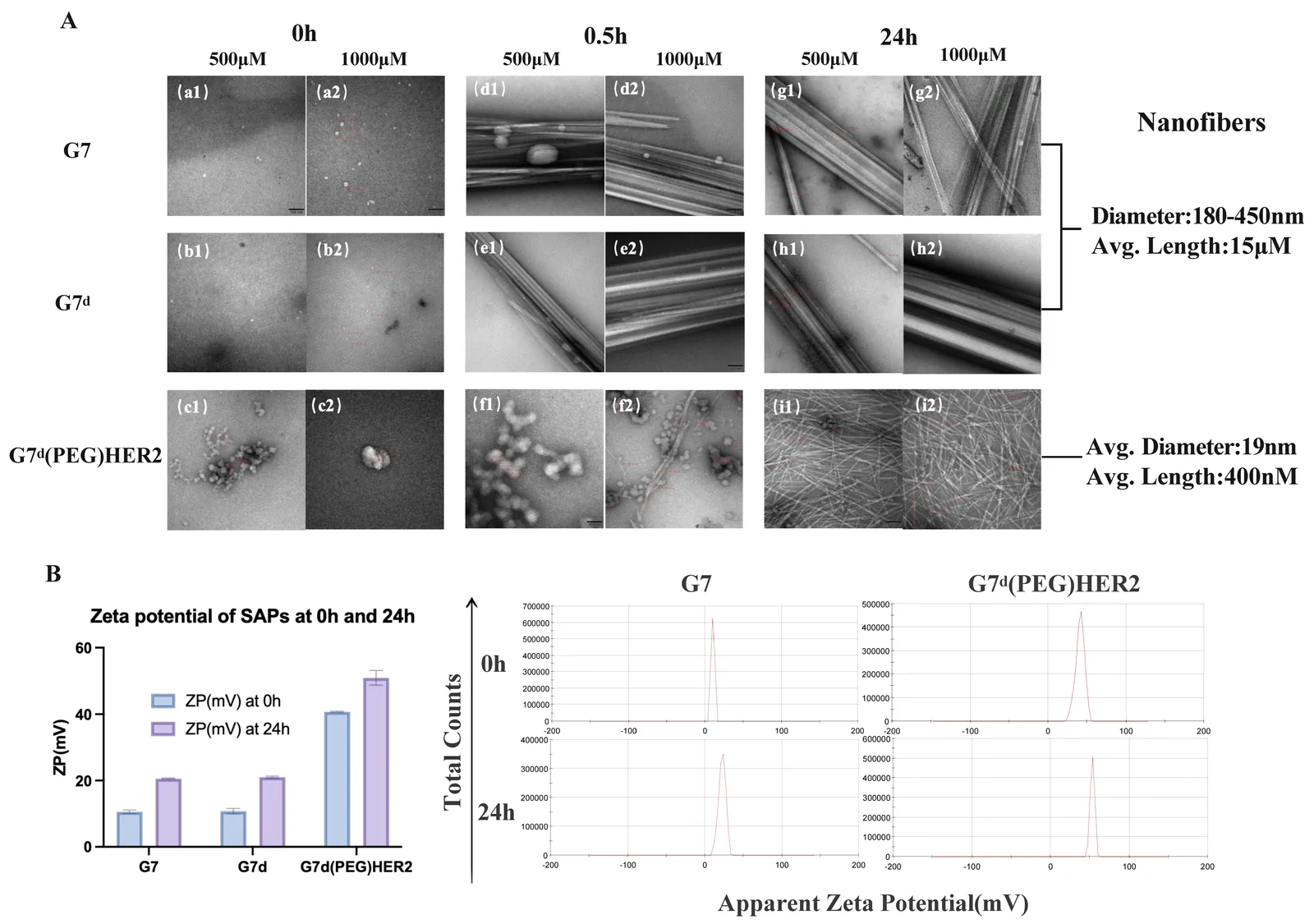
Characterizations of the self-assembly peptides including: (**A**) Representative transmission electron microscopy (TEM) images (scale bar = 100 nm); (**B**) zeta potential of peptides suspended in PBS.

In this study, we used peptides including G7 and G7d and G7d(PEG)HER2 (Table 1) were acquired from Chinese peptide company with the purity higher than 95% and endotoxin level lower than 5 EU/mg. Material size and morphology before and after self-assemble were characterized by TEM (Figure 1A), zeta potential before and after self-assemble in PBS were measured by NanoZeta sizer. All the peptides were spherically shaped particles before self-assemble with a primary diameter of about ~20 nm. All the peptides assembled after 24 h under room temperature at the concentration of 500 μM and 1000 μM. G7 and G7d assembled into nano crystals with a diameter ranging from 180–450 nm and a length of 15 μm, tend to aggregate into clusters, exhibiting poor dispersibility. G7d(PEG)HER2 self-assembles into nanofibers with a diameter of 19 nm and a length of 400 nm, showing uniform dispersion. The zeta potentials of all peptides suspended in PBS were positive, G7d(PEG)HER2 exhibited the highest absolute zeta-potential value (Figure 1B).

### 3.2. Peptide Nanomaterials Reduced Viability of Immune Cells Especially Macrophages

**Figure 2:**
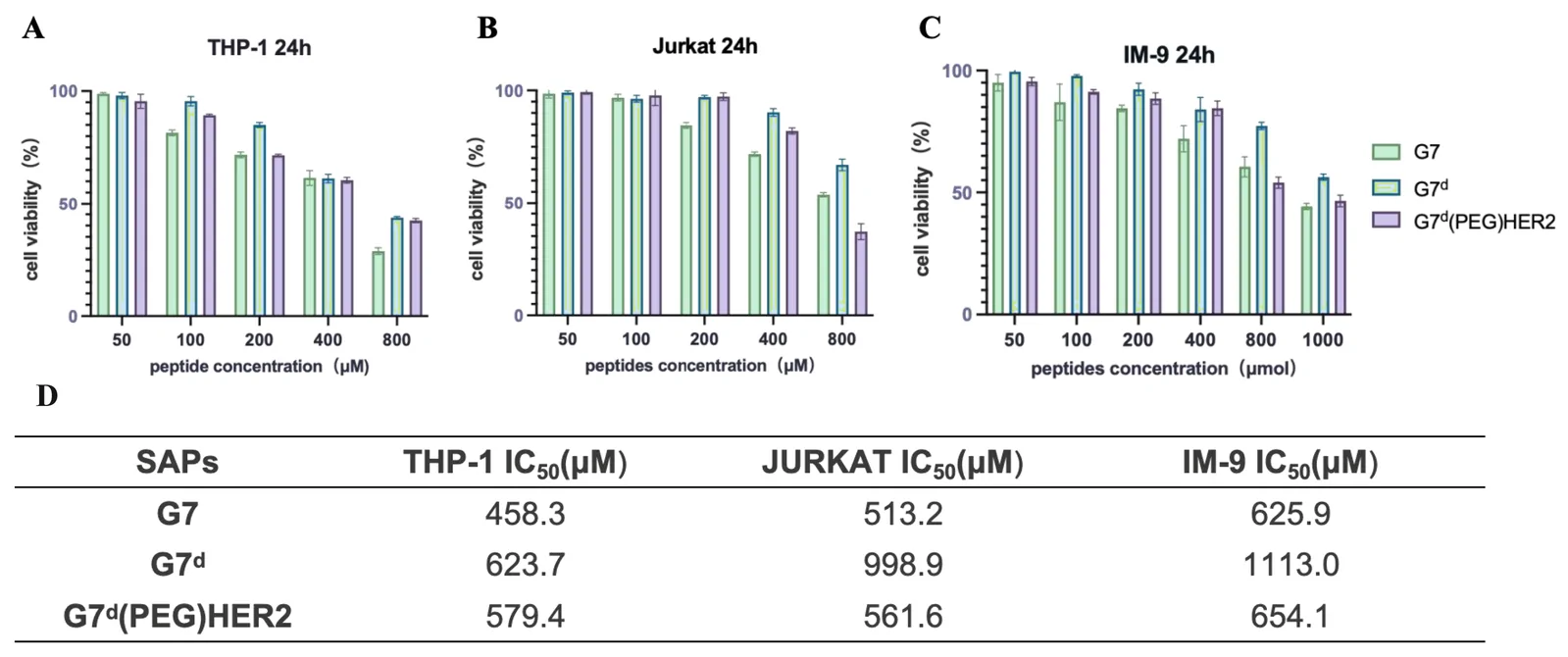
IC50 of THP-1, Jurkat clone E6-1 and IM-9 cells after 24 h exposure of peptides. (**A**) cell viability changes of THP-1 cells after peptides treatment under 50 μM, 100 μM, 200 μM, 400 μM, 800 μM concentrations of peptides; (**B**) cell viability changes of Jurkat clone E6-1 cells after peptides treatment under 50 μM, 100 μM, 200 μM, 400 μM, 800 μM concentrations of peptides; (**C**) cell viability changes of IM-9 cells after peptides treatment under 50 μM, 100 μM, 200 μM, 400 μM, 800 μM, 1000 μM concentrations of peptides; (**D**) IC50 values. Cell viability is expressed as % change relative to the negative control (blank, no particle exposure; n = 3). Statistical annotations and IC50 variability should be finalized after replicate-level dose-response analysis.

CCK-8 assays were performed on THP-1, JURKAT, and IM-9 cells to determine the half-maximal inhibitory concentration (IC50) values of different peptides. All three SAPs exhibited relatively low cytotoxicity. Among them, G7d showed the weakest inhibitory effect on cell viability and the lowest level of toxicity compared with G7 and G7d(PEG)HER2 (Figure 2). Dose-dependent cellular responses to the Sup35 G7 based self-assembling peptide nanomaterials are shown in Figure 2A–C across the tested concentration ranges in THP-1 based macrophages, Jurkat T cells, and IM-9 B cells. The effects of peptides varied among the three immune cells: peptides had the most pronounced impact on THP-1 cells (Figure 2A,D), whereas their effects on JURKAT (Figure 2B,D) and IM-9 cells (Figure 2C,D) were milder, reflected by higher IC50 values.

### 3.3. Peptide Nanomaterials Mainly Influence Macrophages

#### 3.3.1. Transcriptome Analysis

##### Sequencing Data

On the basis of the off-machine data of 27 samples including PBS, 500 μM G7 and G7d(PEG)HER2 treated 3 cell lines for 24 h, each performed in triplicates. After data filtering, we finally obtained a total of 194.23 G Clean bases. For each sample, bases with Phred values greater than 30 accounted for an average of 96.14%, bases with Phred values greater than 20 accounted for an average of 99.05%.

##### Analysis of Defferentially Expressed Genes

**Figure 3:**
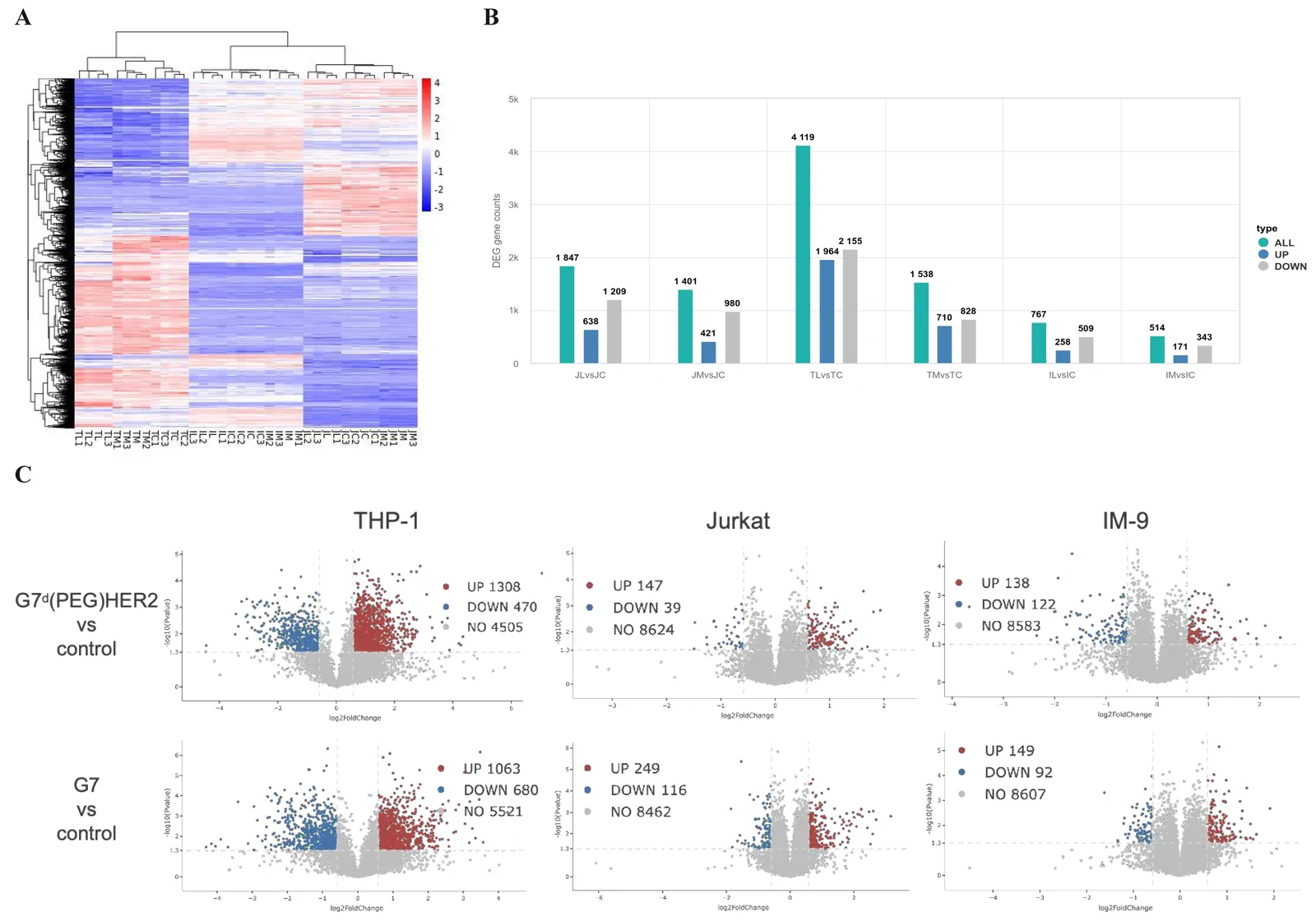
Transcriptome and proteome analysis. (**A**) heatmap of DEGs cluster analysis; (**B**) Bar graph of DEGs analysis; (**C**) volcano graph of DEPs analysis.

This study screened the 500 μM G7 and G7d(PEG)HEr2 treated THP-1cells, Jurkat clone E6-1 cells and IM-9 cells groups according to the criteria of |log2(fold change)| ≥ 1 and *p*adj ≤ 0.05, revealing a total of 10,186 DEGs. Among these, in THP-1 cells, 2155 DEGs were downregulated, 1964 DEGs were upregulated in G7 treated group, 828 DEGs were downregulated, 710 DEGs were upregulated in G7d(PEG)HER2 group; in Jurkat clone E6-1 cells, 1209 DEGs were downregulated, 638 DEGs were upregulated in G7 treated group, 980 DEGs were downregulated, 421 DEGs were upregulated in G7d(PEG)HER2 group; in IM-9 cells, 509 DEGs were downregulated, 258 DEGs were upregulated in G7 treated group, 343 DEGs were downregulated, 171 DEGs were upregulated in G7d(PEG)HER2 group (Figure 3A,B).

#### 3.3.2. Proteome Analysis

##### Analysis of Differential Expressed Proteins (DEPs)

This study used DIA mode to carry out quantitative proteomics research and identified a total of 101,242 peptides and 10,631 proteins in 27 samples. In THP-1 cells, a total of 470 DEPs were significantly downregulated, 1308 DEPs were significantly upregulated in G7^d^(PEG)HER2 treated group, 680 DEPs werte significantly downregulated, 1063 DEPs were significantly upregulated in G7 treated group; in Jurkat clone E6-1 cells, a total of 39 DEPs were significantly downregulated, 147 DEPs were significantly upregulated in G7^d^(PEG)HER2 treated group, 116 DEPs werte significantly downregulated, 249 DEPs were significantly upregulated in G7 treated group; in IM-9 cells, a total of 122 DEPs were significantly downregulated, 138 DEPs were significantly upregulated in G7^d^(PEG)HER2 treated group, 92 DEPs werte significantly downregulated, 149 DEPs were significantly upregulated in G7 treated group (Figure 3C).

#### 3.3.3. Joint Proteome-Transcriptome Analysis

**Figure 4:**
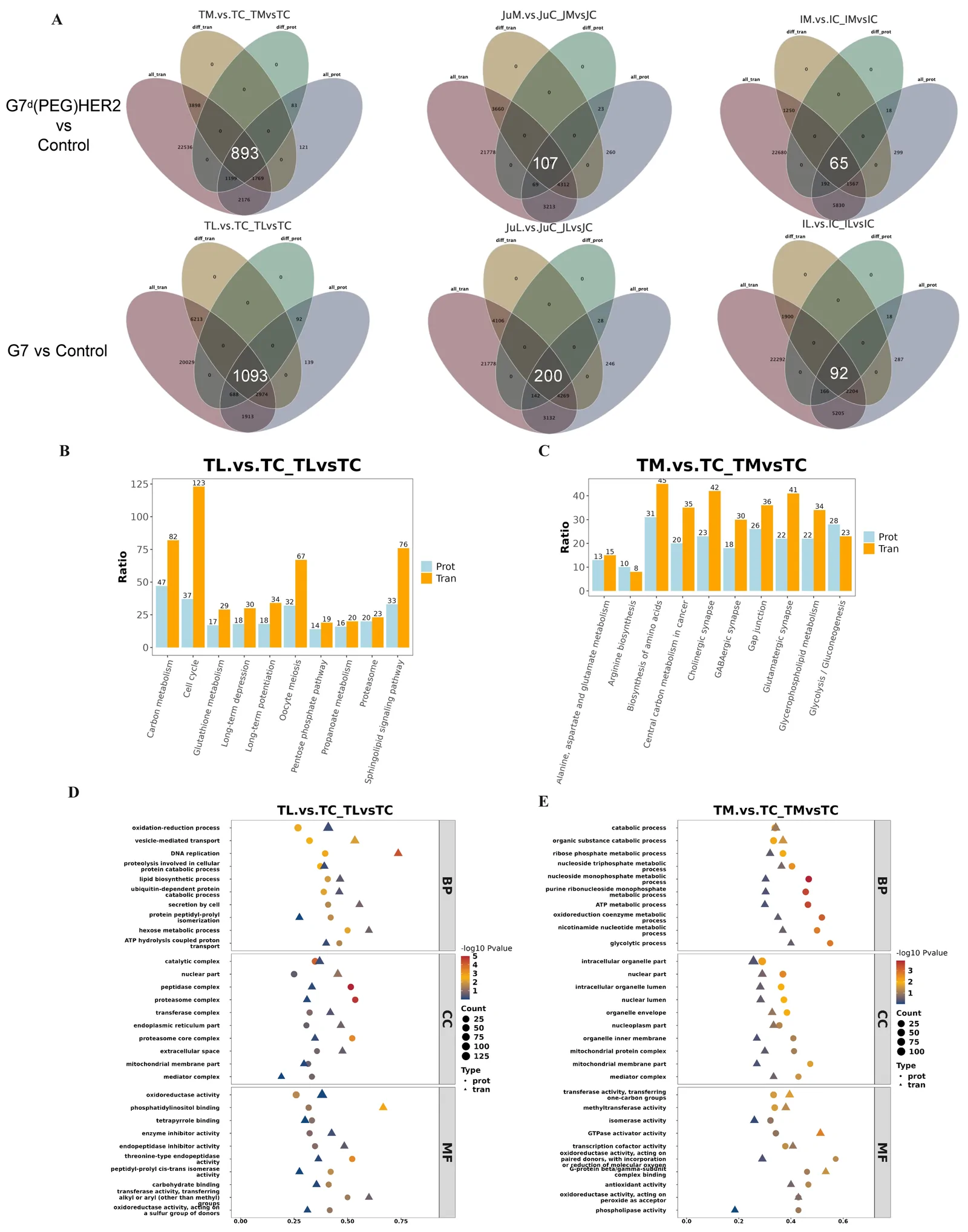
Combined transcriptome and proteome analysis. (**A**) co-expressed genes; (**B**,**C**) KEGG enrichment; (**D**,**E**) GO enrichment.

By combining proteomics and transcriptomics, gene expression under different treatments can be studied. By integrating mRNA and protein information, 2540 co-expressed genes were identified in total. In THP-1 cells, 893 co-expressed genes were identified in G7^d^(PEG)HER2 treated group, 1093 co-expressed genes were identified in G7 treated group; in Jurkat clone E6-1 cells, 107 co-expressed genes were identified in G7^d^(PEG)HER2 treated group, 200 co-expressed genes were identified in G7 treated group; in IM-9 cells, 65 co-expressed genes were identified in G7^d^(PEG)HER2 treated group, 92 co-expressed genes were identified in G7 treated group (Figure 4A).

##### KEGG and GO Analyses of the Shared DEGs and DEPs in THP-1 Cells

Through functional annotation of common DEGs and DEPs, the results were divided into four categories: upregulated expression in both the transcriptome and proteome and downregulated expression in both the transcriptome and proteome. The transcriptome expression was downregulated, the transcriptome expression was upregulated, and the proteome expression was downregulated.

KEGG analysis (Figure 4B,C) revealed that the major metabolism pathways including cell cycle, carbon metabolism, sphingolipid signaling pathway, oocyte meiosis, long-term potentiation, long-term depression, glutathione metabolism, proteasome, propanoate metabolism and pentose phosphate pathways were upregulated in both the transcriptome and proteome in the G7 treated group, while biosynthesis of amino acids, cholinergic synapse, clutamergic synapse, gap junction, central carbon metabolism in cancer, glycerophpsphlipid metabolism, GABAergic synapse, glycolysis/gluconeogenesis, alanine, aspartate and glutamate metabolism and arginine biosynthesis pathways were upregulated in both the transcriptome and proteome in the G7^d^(PEG)HER2 treated group (*p* < 0.05).

In the GO analysis (Figure 4D,E) revealed that the expression of both the transcriptome and the proteome was upregulated, the enriched genes associated with biological process (BP) included DNA replication, secretion by cell and hexose metabolic process, the enriched genes associated with cellular component (CC) included catalytic complex, peptidase complex, proteasome core complex and mitochondrial membrane part, the enriched genes associated with molecular function (MF) included phosphatiadylinositol binding and threonine-type endopeptidase activity in G7 treated group; the enriched genes associated with BP included catabolic process, organic substance catabolic process, nucleoside monophosphate metabolic process, ATP metabolic process, oxidoreduction coenzyme metabolic process and glycolytic process, the enriched genes associated with CC included intracellular organelle part, nucleoplasm part and mediator complex, the enriched genes associated with MF included transferase activity, transferring one-carbon groups, methytransferase one-carbon groups, methyltransferas activity, GTPase activity, acting on paired donors, with incorporation or reduction of molecular oxygen, G-protein beta/gamma-subunit complex binding, antioxidant activity and oxidoreductase activity, acting on peroxide as acceptor in G7^d^(PEG)HER2 treated group (*p* < 0.05).

### 3.4. Interactions Between Peptide Nanomaterials with Macrophages

#### 3.4.1. Cellular Uptake of Peptides in Macrophages

**Figure 5:**
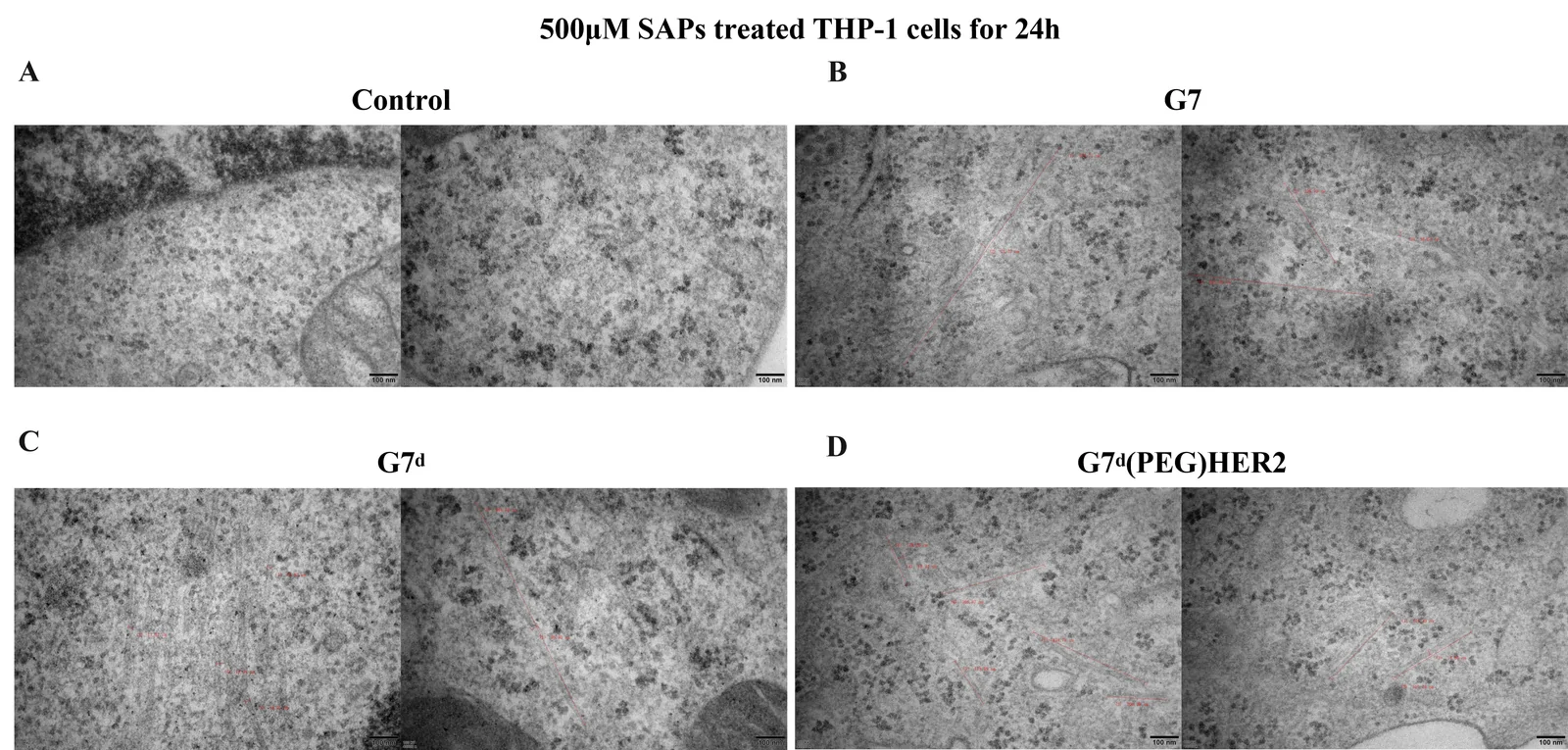
Cellular uptake of SAPs in macrophages observed under TEM (scale bar = 100 nm). (**A**) Control group treated by PBS; (**B**) 500 μM G7 treated group; (**C**) 500 μM G7d treated group; (**D**) 500 μM G7d(PEG)HER2 treated group.

Due to our previous results, all three self-assembly peptides carry positive surface charge which is believed can exhibit the most pronounced effects on macrophages. Nanomaterials with a positive zeta potential are primarily internalized by cells through multiple pathways, including endocytosis. Therefore, we further analyzed the uptake of peptides by THP-1 cells. After treating THP-1 cells with three types of peptides at a concentration of 500 μM for 24 h, fully self-assembled SAPs were observed in the cytoplasm, and marked mitochondrial shrinkage was detected in some cells (Figure 5).

#### 3.4.2. Sub-Lethal Effects After Peptides Treatment

**Figure 6:**
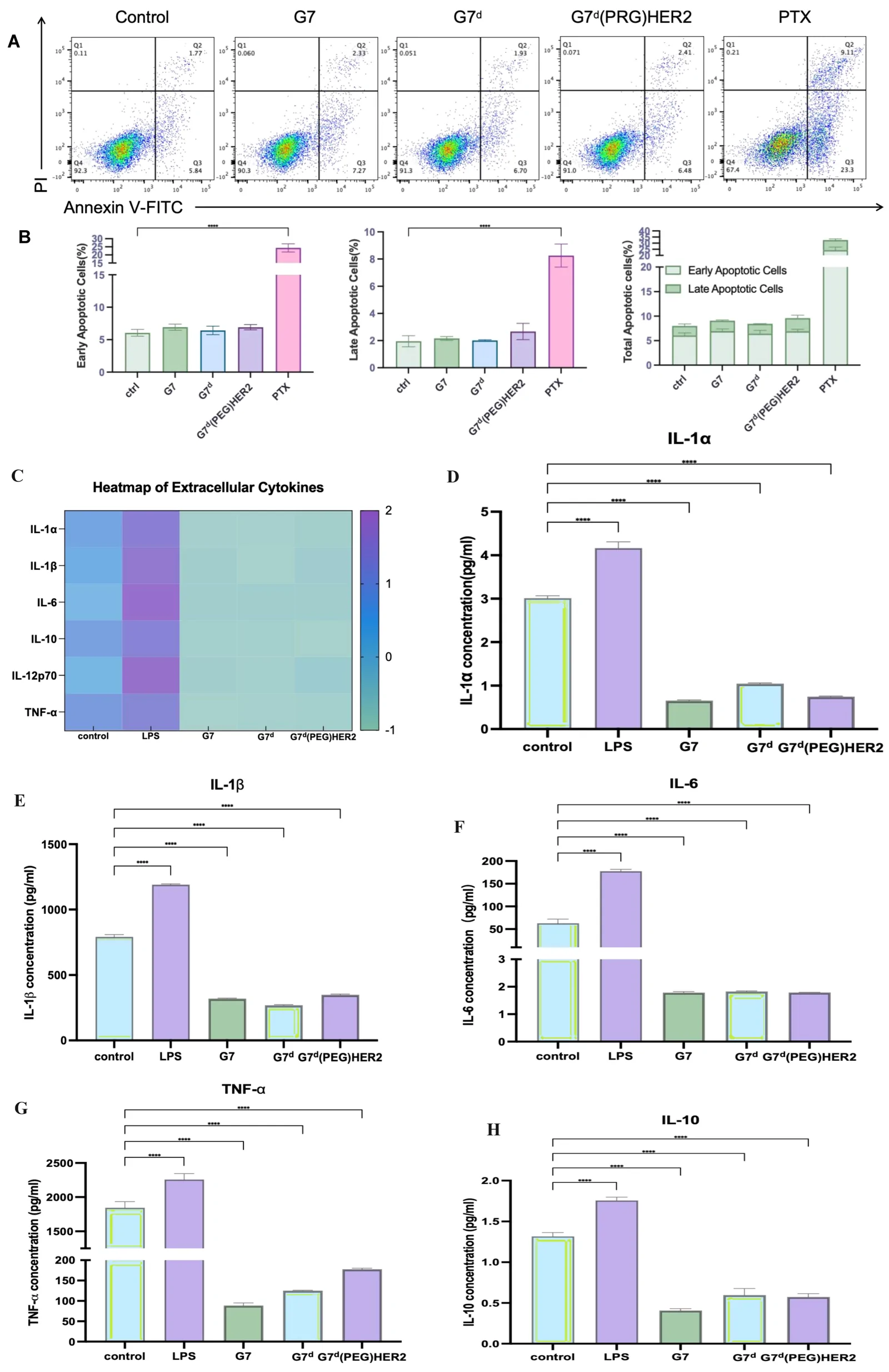

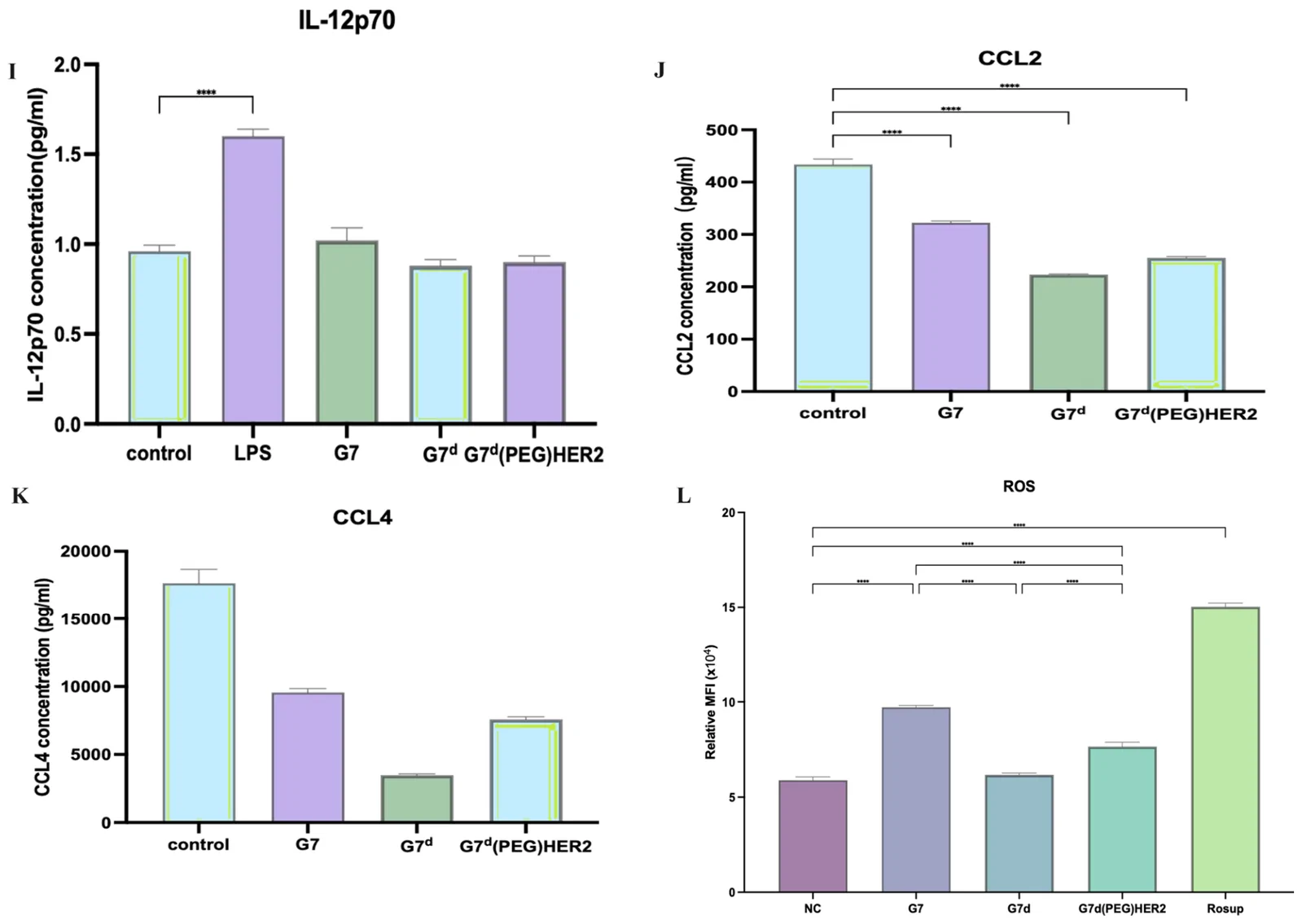
Impacts of SAPs on macrophages. (**A**,**B**) Apoptosis test of THP-1 cells after 24 h treatment of PBS (negative control), PTX (Paclitaxel, positive control), G7, G7^d^ and G7^d^(PEG)HER2 under 500 μM concentration, cells were stained by annexin v and PI; (**C**) Heatmap of extracellular inflammatory cytokines induced by 500 μM G7, G7d and G7^d^(PEG)HER2 based on Luminex assay. The data in the heatmap represent the changes in secretion of cytokines caused by the SAPs, compared to the control group. Purple indicates increased cytokine secretion, whereas green indicates decreased cytokine secretion relative to the control group. (**D**–**L**) **** *p* < 0.0001.

Due to the results above, we try to find out the underlying mechanism how SAPs impacting macrophages. Flow cytometric analysis revealed that treatment with all three self-assembling peptides did not result in detectable apoptosis in THP-1 cells (Figure 5A,B). To characterize cellular inflammatory responses following particle exposure, cytokines were measured using Luminex assay. For pro-inflammatory markers (IL-1α, IL-1β, IL-6, IL-10, IL-12p70, CCL2, CCL4), significant decreases from negative control were observed following peptides exposures (Figure 6C–K). Oxidative stress has been recognized as a key mechanism underlying nanomaterials toxicity. This study confirmed that G7 may induce intracellular reactive oxygen species (ROS) production compares to G7^d^ and G7^d^(PEG)HER2 from DCF assay (Figure 6L).

### 3.5. In Vivo Humoral Immune Responses Induced by SAPs

**Figure 7:**
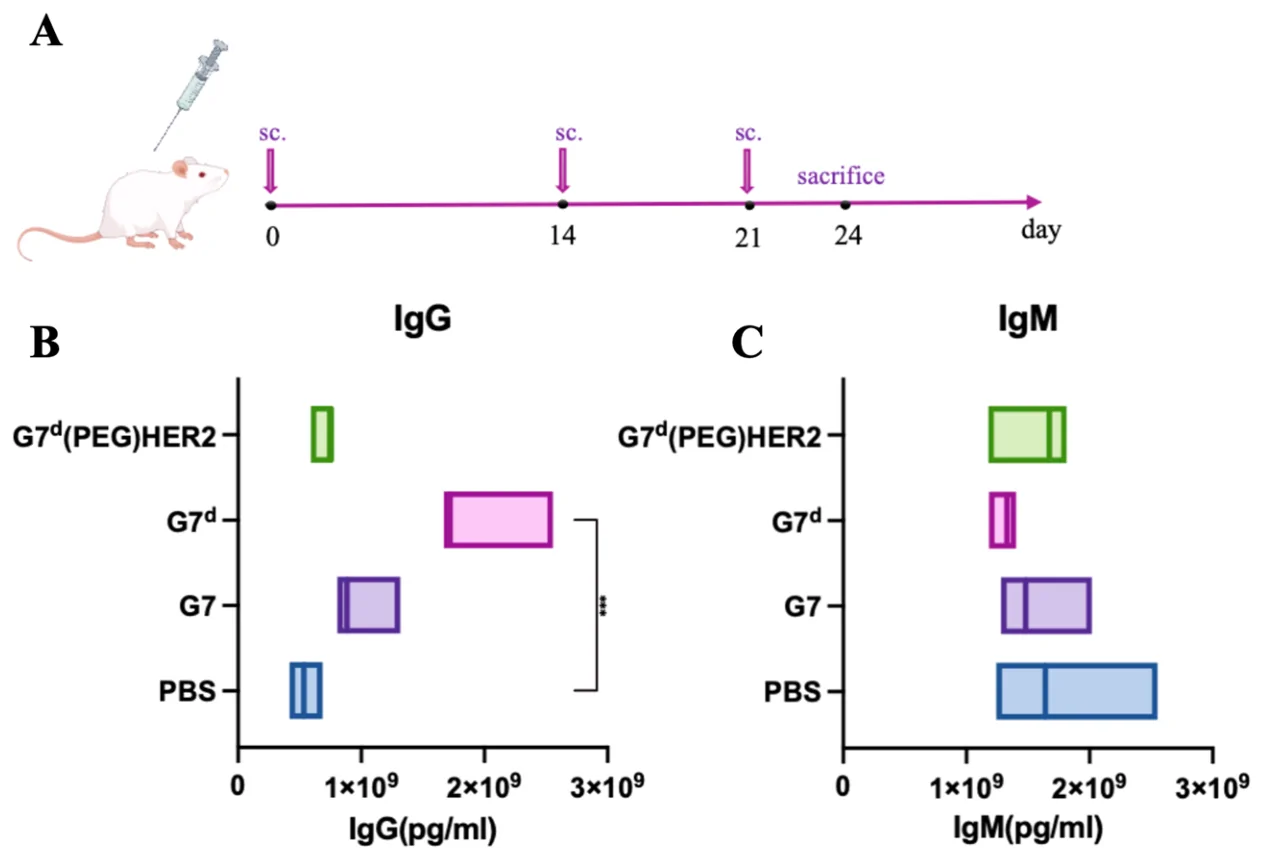
Impacts of SAPs on IgG and IgM in health female Balb/c Mice serum. (**A**) Scheme of in vivo immunization with 20 mg/kg SAPs; (**B**) Changes of IgG; (**C**) Changes of IgM. n = 5. Statistical annotations should be finalized after one-way ANOVA followed by an appropriate post-hoc multiple-comparison test. *** *p* < 0.001.

To further evaluate the in vivo immune compatibility of the Sup35 G7 based self-assembling peptide nanomaterials, healthy female BALB/c mice were subcutaneously immunized with G7, G7d, or G7d(PEG)HER2 at 20 mg/kg on days 0, 14, and 21, and serum samples were collected on day 24 (Figure 7A). Serum immunoglobulin levels were determined using a Luminex-based antibody isotyping assay. Compared with the PBS control group, G7d treatment increased serum IgG levels, whereas G7 showed a smaller change and G7d(PEG)HER2 showed no evident enhancement of IgG production (Figure 7B). The statistical interpretation of pairwise group differences should be finalized using an appropriate post-hoc multiple-comparison test. In contrast, serum IgM levels did not show a significant increase after treatment with any of the three peptide nanomaterials compared with PBS control (Figure 7C). These results suggest that repeated immunization of G7d preferentially promotes an IgG-dominant humoral response, whereas PEGylated G7d(PEG)HER2 exhibits a comparatively weaker systemic antibody response under the present experimental conditions.

## 4. Discussion

In this study, we systematically investigated the structure-dependent interactions between Sup35 G7 based self-assembling peptide nanomaterials and representative human immune cell models, with particular emphasis on macrophage responses. By combining physicochemical characterization, cytocompatibility assessment, transcriptomic and proteomic profiling, and functional validation, we found that these peptide assemblies exhibited low acute cytotoxicity but induced measurable macrophage responses involving metabolic remodeling, redox regulation, and mitochondrial-associated alterations. These findings suggest that Sup35 G7 based SAPs are not simply inert peptide materials; rather, their assembly morphology and surface modification can shape innate immune-cell responses in a structure-dependent manner. This point is particularly relevant for the safety-oriented design of peptide-based nanovaccine scaffolds, where controlled interaction with innate immune cells is required without excessive cytotoxicity or uncontrolled inflammatory activation.

### 4.1. Structural Features Govern Immune-Cell Interaction Patterns

Our results demonstrate that self-assembly morphology and surface modification are key determinants of cellular responses. As shown by TEM (Figure 1), G7 and G7^d^ form large crystalline aggregates with poor dispersibility, whereas G7^d^(PEG)HER2 assembles into uniformly distributed nanofibers. These structural differences are expected to influence sedimentation behavior, effective cellular exposure, and membrane interaction.

All materials exhibit positive surface charge, which likely enhances electrostatic interactions with negatively charged cell membranes [20]. This is consistent with the observed preferential uptake in macrophages, supporting the notion that physicochemical parameters directly shape innate immune recognition and internalization efficiency, which is critical in the context of nanovaccine delivery systems.

The in vivo immunization experiment provides additional evidence that the immune effects of Sup35 G7 based SAPs are structure-dependent. Among the three materials, G7d induced the most pronounced increase in serum IgG, while IgM remained largely unchanged. This pattern suggests that repeated exposure to G7d may favor a class-switched humoral response rather than an early IgM-dominant response. In contrast, G7d(PEG)HER2 showed limited induction of serum IgG and IgM, which may be related to its PEG modification and more uniformly dispersed nanofibrous morphology. PEGylation can alter biodistribution.

### 4.2. Macrophages as Primary Responders to Peptide Nanomaterials

Across all assays, macrophages showed the most significant responses compared with Jurkat T cells and IM-9 B cells. This is in line with their intrinsic phagocytic capacity and their role as first-line sensors of particulate materials.

Importantly, although cell viability remained relatively high, multi-omics data revealed substantial transcriptional and proteomic changes specifically in THP-1 based macrophages. This indicates that subtle but coordinated cellular reprogramming can occur in the absence of overt cytotoxicity, highlighting the necessity of using molecular-level analyses when evaluating nanomaterial immune compatibility.

### 4.3. Metabolic and Redox Remodeling Dominates over Inflammatory Activation

Integrated transcriptomic and proteomic analyses consistently showed enrichment of pathways related to energy metabolism, redox regulation, proteasome activity, and mitochondrial function. In contrast, classical inflammatory signaling pathways were not prominently enriched.

This observation is supported by functional data showing no detectable apoptosis and the decreased secretion of multiple cytokines and chemokines (IL-1α, IL-1β, IL-6, IL-12p70, CCL2, CCL4).

Together, these findings suggest that the dominant cellular response is metabolic and functional modulation rather than pro-inflammatory activation. Reduced cytokine levels do not necessarily indicate immunosuppression but may reflect altered cellular activity states or metabolic constraints [21].

### 4.4. Mitochondrial Involvement and Oxidative Stress as Key Mechanisms

We have observed mitochondrial shrinkage following peptide exposure, indicating organelle-level stress. At the same time, G7 induced higher intracellular ROS levels compared with G7^d^ and G7^d^(PEG)HER2, which is consistent with enrichment of glutathione metabolism and pentose phosphate pathways.

These results collectively support a mechanism in which mitochondrial perturbation and redox imbalance act as central mediators of macrophage responses to peptide assemblies. The absence of apoptosis despite mitochondrial alterations further indicates that these effects are sublethal and potentially reversible. PEGylation and nanofiber formation may alter protein corona formation, uptake kinetics, and intracellular trafficking, thereby modulating cellular stress responses [22].

### 4.5. Implications for Vaccine-Oriented Peptide Nanomaterial Assessment

For peptide-based nanovaccine design, candidate scaffolds should ideally support interaction with innate immune cells while avoiding excessive cytotoxicity, uncontrolled inflammation, or unintended immune suppression [23]. The present study does not evaluate antigen loading, antigen presentation, antigen-specific adaptive immune responses, or long-term formulation stability, and the tested materials were not designed as final vaccine formulations. Instead, it provides an early-stage immune compatibility framework for assessing Sup35 G7 based SAPs as vaccine delivery peptide nanomaterials. From this perspective, the results have three implications. First, conventional viability testing is insufficient for evaluating candidate nanomaterial scaffolds, because substantial macrophage molecular remodeling may occur without overt cell death. Second, macrophage metabolic and redox responses should be considered when assessing the safety and immunological suitability of self-assembling peptide materials. Third, assembly morphology and surface modification can shift the balance between cellular uptake, oxidative stress, and cytokine regulation. These structure–immune relationships may inform the rational design of peptide-based nanomaterial scaffolds with more predictable innate immune profiles.

### 4.6. Limitations

- Our study was conducted using immortalized cell lines, which may not fully represent primary immune cell behavior. The present study did not include validation in primary bone marrow based dendritic cells or macrophages; therefore, the cellular findings require confirmation in primary murine innate immune cells. In addition, omics analyses were performed at a single concentration and time point, which limits interpretation of dose-response relationships beyond the dose-dependent viability data. Hydrodynamic size, PDI, and 15-day DLS-based colloidal stability at different temperatures were not determined in this study; therefore, the present data should not be interpreted as evidence of final vaccine formulation stability.
- Future studies should include primary human macrophages, in vivo validation on disease model, and evaluation of antigen-loaded systems to better define the translational potential of these materials in vaccine delivery applications.

## 5. Conclusions

In summary, Sup35 G based self-assembling peptide nanomaterials exhibit low acute cytotoxicity but induce distinct, structure-dependent responses in macrophages, which are more pronounced than in T and B lymphocyte models.

Integrated transcriptomic, proteomic, and functional analyses indicated that these responses were primarily characterized by metabolic remodeling, redox regulation, and mitochondrial-associated alterations, rather than overt inflammatory activation or apoptotic cell death.

Differences in assembly morphology and surface modification influenced intracellular oxidative stress and macrophage response patterns. In particular, G7 was associated with stronger ROS induction, whereas PEGylated G7d(PEG)HER2 displayed a distinct response profile consistent with the influence of surface modification and nanofiber formation.

In vivo immunization results further suggest that these structural differences may extend to systemic immune outcomes, as G7d was associated with an increase in serum IgG levels, while IgM responses remained limited across groups. However, these findings reflect general humoral responses rather than antigen-specific immunity. Overall, this study provides experimental evidence supporting a structure–immune interaction framework for Sup35 G7-derived self-assembling peptides. These findings underscore the importance of controlling physicochemical properties when developing peptide-based nanomaterials as candidate vaccine nanomaterials.

## Figures and Tables

**Table 1:** Self-assembly peptides amino acid sequences, molecule weight, purity and endotoxin.

Self-Assembly Peptides	Sequence	Molecule Weight	Purity	Endotoxin
G7	Gly-Asn-Asn-Gln-Gln-Asn-Tyr	836.8	≥95%	≤5 EU/mg
G7^d^	DGly-DAsn-DAsn-DGln-DGln-DAsn-DTyr	836.8		
G7^d^(PEG)HER2	Ac-DLys-His-His-His-Gly-Asn-Asn-Gln-DGln-Asn-Tyr-PEG4-DTyr-Cys-Asp-Gly-Phe-DTyr-Ala-Cys-DTyr-Met-Asp-Val-NH2	3096.3		

## Data Availability

We conform with the Publication Ethics Guidelines. The data supporting the findings of this study are contained within the article.
